# Yoga exercises can reduce prenatal maternal stress

**DOI:** 10.1192/j.eurpsy.2021.1056

**Published:** 2021-08-13

**Authors:** S. Kiselev

**Affiliations:** Clinical Psychology, Ural Federal University, Ekaterinburg, Russian Federation

**Keywords:** pregnancy-specific anxiety, yoga exercises, prenatal maternal stress

## Abstract

**Introduction:**

Prenatal maternal stress is an important phenomenon. Evidence on this topic suggests that women who experience high stress during pregnancy are more likely to deliver preterm infants.

**Objectives:**

The goal of this study was to evaluate the influence of Yoga exercises training on stress reduction during pregnancy.

**Methods:**

In the current study we included 20 women who participated in the Yoga exercises training during pregnancy. The control group included 20 women who were in the reading control condition during pregnancy. Women were eligible to participate if they were experiencing elevated levels of perceived stress or pregnancy-specific anxiety (PSA), as indicated by responses to the Perceived Stress Scale and the PSA scale on a screening questionnaire. Women enrolled between 12 and 26 weeks gestation were randomly assigned to either the Yoga exercises training or to the reading control condition. Effects of training were analyzed by means of an ANOVA with repeated measurements.

**Results:**

ANOVA has revealed (p<.05) that women in the Yoga exercises training experienced larger decreases from pre- to postintervention in pregnancy-specific anxiety and pregnancy-related anxiety than participants in the reading control condition.

**Conclusions:**

This pilot study suggests that Yoga exercises training during pregnancy can effectively reduce pregnancy-related anxiety. However, it is necessary to do further research on the impact of Yoga exercises on stress reduction during pregnancy.

